# Bradycardia in Patients With COVID-19: A Calm Before the Storm?

**DOI:** 10.7759/cureus.8599

**Published:** 2020-06-13

**Authors:** Eluwana A Amaratunga, Douglas S Corwin, Lynn Moran, Richard Snyder

**Affiliations:** 1 Internal Medicine, St. Luke’s University Health Network, Easton, USA; 2 Pulmonary and Critical Care, St. Luke’s University Health Network, Easton, USA; 3 Cardiology, St. Luke’s University Health Network, Easton, USA

**Keywords:** covid-19, corona virus disease, sars-cov-2 (severe acute respiratory syndrome coronavirus -2), arrhythmia, bradycardia, cytokine release syndrome (crs), cardiovascular

## Abstract

Cardiac manifestations of coronavirus disease 19 (COVID-19), including arrhythmia, have been described in the literature. However, to our knowledge, association of COVID-19 with bradycardia has not been reported. This case study describes sinus bradycardia as a potential manifestation of COVID-19.

This is a retrospective case series of four patients with laboratory-confirmed severe acute respiratory syndrome coronavirus 2 (SARS-CoV-2) infection, admitted to St. Luke’s University Health Network ICU between 24 March 2020 and 5 April 2020. Medical records of these patients were reviewed using the EPIC electronic health record system. Demographic, clinical, laboratory, and treatment data were reviewed against periods of bradycardia in each patient.

The patient group comprised two males and two females. Two patients had pre-existing cardiovascular (CV) comorbidities but no history of arrythmias. Heart rates ranged between 66 and 88 beats/min on admission. The lowest rates during bradycardia were between 42 and 49 beats/min. The onset of sinus bradycardia in patients 1, 2, and 3 were day nine, 15, and five of illness, respectively. Patient 4 had three episodes of bradycardia, starting on day 10 of illness. Patients’ bradycardia episodes lasted one to 14 days. During bradycardia, maximum body temperatures ranged between 99.9 and 100.2 degree Fahrenheit. Patients 2, 3, and 4 required vasopressors to maintain mean arterial pressure > 65 mmHg during episodes. All four patients were on propofol at some point during bradycardia with patients 1, 2, and 3 also receiving dexmedetomidine. There was no consistent correlation of these medications with bradycardia. Electrocardiogram (ECG) findings included sinus bradycardia. Prolonged QTc interval observed in patient 2 on admission improved during bradycardia.

Transient sinus bradycardia is a possible manifestation of COVID-19 and is important for close CV surveillance. Etiology can be multifactorial, but severe hypoxia, inflammatory damage of cardiac pacemaker cells, and exaggerated response to medications are possible triggers. High levels of pro-inflammatory cytokines may act directly on the sinoatrial (SA) node contributing to the development of bradycardia. This may be a warning sign of the onset of a serious cytokine storm. An increased awareness of possible exaggerated bradycardia response is important to consider with the use of empiric medications which have arrhythmogenic effects.

## Introduction

In December 2019, the first coronavirus disease 19 (COVID-19) patient was reported in Wuhan, China. Since then, the severe acute respiratory syndrome-coronavirus 2 (SARS-CoV-2) has rapidly spread reaching pandemic status within a few months. While ongoing research efforts are providing a vast amount of information, there is much regarding this novel coronavirus that remains unknown. Cardiac manifestations of COVID-19 have been reported with a higher prevalence described in patients admitted to ICUs [1-2]. He et al. reported two patients with COVID-19 with different electrocardiographic (ECG) manifestations [3]. The first patient was a previously healthy 66-year-old female who demonstrated a transient S1Q3T3 finding on the day of tracheal intubation, followed by reversible nearly complete atrioventricular (AV) block. A simultaneous echocardiogram performed demonstrated findings of elevated pulmonary artery pressure. The other patient reported was a 77-year-old male with a history of hypertension and type 2 diabetes, who required veno-venous extracorporeal membrane oxygenation (VV-ECMO) for severe hypoxia, without significant improvement in his oxygenation. The ECG findings in this patient included ST segment elevation accompanied by multifocal ventricular tachycardia, with an increase in levels of cardiac troponin I (cTnI) [3].

Acute cardiac injury (elevation of cTnI above 99th percentile upper reference limit) is the most commonly reported cardiac complication in COVID-19, affecting approximately 8%-12% of all patients with COVID-19 [4]. Other reported clinical manifestations include acute coronary events, acute left ventricular (LV) systolic dysfunction, acute congestive heart failure, and cardiac arrhythmias [4-6]. A study involving 138 patients infected with COVID-19 in Wuhan, China, demonstrated cardiogenic shock, arrhythmia, and acute cardiac injury to be among common complications; 8.7%, 16.7%, 7.2% respectively [1]. The incidence of arrhythmia was higher in patients admitted to the ICU compared to those who were not [4].

Bradycardia is another cardiac manifestation of COVID-19 that has not been previously reported in the medical literature. This clinical sign was noted in several patients receiving care in our ICU. While there have been a few studies regarding the development of tachy- and bradyarrhythmias in patients with COVID-19, the specific nature of the dysrhythmias was not reported [1, 7]. A recent publication stated that tachycardia might be a common arrhythmia in patients with COVID-19 [8].To the best of our knowledge, and in review of medical literature, this is the first time that there has been an association with COVID-19 and bradycardia.

## Case presentation

Method

Study Design and Participants

This is a retrospective case series involving four patients admitted to St. Luke's University Health Network - Anderson Campus ICU between 24 March 2020 and 5 April 2020. All four patients were confirmed positive for COVID-19 pneumonia with severe acute hypoxic respiratory failure requiring intubation and mechanical ventilation. A positive confirmation of COVID-19 was determined by the detection of SARS-CoV-2 in polymerase chain reaction (PCR) of nasopharyngeal specimens.

Data Collection

The EPIC electronic health record system was used to review medical records of each patients’ hospital course. Patient demographics, comorbidities, presenting day of illness since symptom onset, admission heart rate, duration of illness at intubation, duration of illness at onset of bradycardia, vital signs [blood pressure, mean arterial pressure (MAP), oxygen saturation, respiratory rate, temperature], laboratory studies (including cTnI, ferritin, C-reactive protein, D-dimer, fibrinogen), as well as medications and dose adjustments were investigated and compared against episodes of bradycardia. The ECGs acquired on admission and during bradycardic episodes were reviewed to further characterize the bradycardia. Electronic cardiac monitoring was reviewed as well.

Results

Baseline characteristics of patients (1-4) at hospital admission are presented in Table 1. Their ages were 55, 60, 78, and 73 years, respectively. Two patients were of male gender and the other two female. Patients 1 and 2 had no documented cardiovascular (CV) comorbidities. Patients 3 and 4 had underlying coronary artery disease (CAD), hypertension (HTN), and hyperlipidemia (HL). None had previous history of either brady- or tachy-arrhythmias. Their heart rates on admission ranged between 66 and 82 beats/min. They presented to the hospital more than five days since symptom onset and required intubation and ventilation within one day of admission due to acute hypoxic respiratory failure.

**Table 1:** Baseline characteristics of patients on admission. CAD, coronary artery disease; HTN, hypertension; HLD, hyperlipidemia; AS, aortic stenosis

Variable	Patient 1	Patient 2	Patient 3	Patient 4
Age (years)	55	60	78	73
Gender	Male	Female	Female	Male
Comorbidities: noncardiac	Hypothyroidism	None	Hypothyroidism	None
Comorbidities: cardiac	None	None	CAD, HTN, HLD	CAD, HTN, HLD, AS
BMI (kg/m^2^)	34.3	24	29.9	35
Presenting day of illness (since admission)	9	11	5	7
Heart rate on admission (beats/min)	82	68	66	76

Development of Sinus Bradycardia During Hospital Admission

Patient 1 developed sinus bradycardia on day nine of illness (day one of hospital admission) and patient 2 on day five of illness (day one of hospital admission). Their bradycardia lasted for 24 hours. Patient 2 developed bradycardia on day 15 of her illness (four days into admission) and persisted for four days until spontaneous resolution. Patient 4 had multiple episodes of bradycardia; days 10-11 (four days into admission), days 13-14, and days 16-18 of illness.

Association of Heart Rate With Body Temperature, Blood Pressure, and Oxygen Saturation

Patients’ maximum body temperatures (Tmax) ranged between 99.9 and 100.2 degree Fahrenheit during bradycardic episodes. Patients maintained MAP >65 mmHg during bradycardia, however, some required vasopressors. All patients except patient 1 were on either norepinephrine or vasopressin since admission. Norepinephrine infusion in patient 2 was increased two hours after onset of bradycardia to maintain a MAP >65 mmHg. Afterward, her infusion rate was gradually decreased, and discontinued two days after bradycardia resolution. Patient 3 was started on norepinephrine two hours prior to bradycardic episode, however, she did not require continuation of the medication. Therefore, infusion was discontinued within the first hour of bradycardia onset. Norepinephrine was re-started on day two of bradycardia due to low MAP and weaned off following bradycardia resolution. Patient 4 was started on norepinephrine and vasopressin two days prior to onset of bradycardia. Vasopressin was continued at the same dose during first bradycardia episode, through day one of the second bradycardia episode. His norepinephrine was at 18 mcg/min three hours before onset of bradycardia, and it was gradually weaned off on day one of bradycardia. He did not require dose increase of norepinephrine in subsequent bradycardic episodes. Patient 1 maintained MAP >65 mmHg during bradycardia without requiring vasopressors.

Medication Effects on Heart Rate

**Figure 1:**
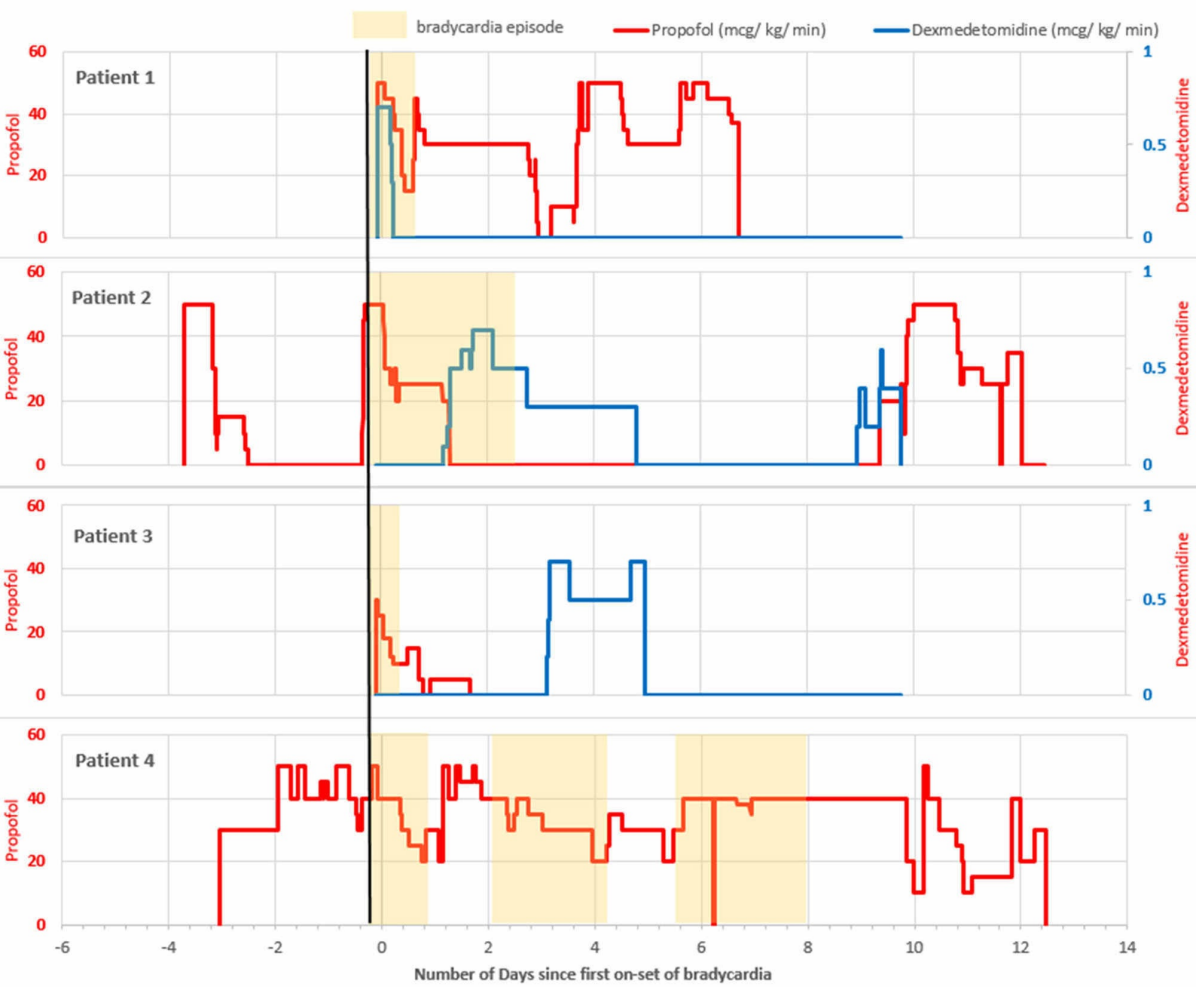
Propofol and dexmedetomidine infusion in relation to bradycardia.

All four patients were on propofol at one point during bradycardia. Patients 1, 2, and 3 were also given dexmedetomidine. Figure 1 summarizes propofol and dexmedetomidine infusions relative to first onset of bradycardia.

Patient 1 was started on propofol and dexmedetomidine infusions two hours prior to bradycardia onset. Dexmedetomidine was discontinued five hours into bradycardia while propofol was continued at a lower rate. Propofol was continued following bradycardia resolution and further increments of propofol dosage did not result in bradycardia. Patient 2 was on a varying combination of fentanyl, propofol, and hydromorphone for over three days prior to onset of bradycardia. The patient was started on dexmedetomidine a day into the episode and continued infusion following the resolution of bradycardia. Seven days after resolution of bradycardia, dexmedetomidine and propofol were re-initiated without the onset of bradycardia. Propofol infusion was initiated two hours prior to bradycardia onset in patient 3. While propofol rate was gradually decreased, the patient continued to receive infusion following bradycardia resolution. Later initiation of dexmedetomidine did not induce bradycardia. Patient 4 was on continuous propofol infusion for three days prior to onset of bradycardia. During the three episodes of bradycardia, there were changes to propofol dosage, with increments of rates in the interim periods. Propofol was continued at the same rate following resolution of the last bradycardia episode.

Azithromycin and hydroxychloroquine were initiated on the day of admission in patients 1, 3, and 4. Patient 2 only received hydroxychloroquine as she had a corrected QT interval (QTc) of 539 milliseconds (ms) on the day of admission. Patients 1 and 3 developed bradycardia on day one and two of azithromycin and hydroxychloroquine combination treatment. Patient 2 developed bradycardia on day five of hydroxychloroquine therapy, and continued to be bradycardic following discontinuation of the medication. Patient 4 was noted to have bradycardia for two days while on azithromycin and hydroxychloroquine combination. This patient subsequently developed further episodes of bradycardia while off of these medications.

While two patients’ home medications included anti-hypertensives (i.e. lisinopril, losartan, atenolol), all home anti-hypertensives including beta-blockers were held on admission.

Electrocardiogram Findings

Initial ECGs on admission showed normal sinus rhythm, with a heart rate ranging between 71 and 93 beats/min. No arrhythmias were noted. Patient 2 did have a prolonged QTc of 539 ms which persisted, but improved to 491 ms during the bradycardic episodes. All patients had normal sinus rhythm during episodes of bradycardia (i.e. sinus bradycardia).

## Discussion

As previously described, acute myocardial injury was thought to be the most common cardiac manifestation in COVID-19 patients and potential for developing cardiac arrhythmias has been noted in a few studies. To our knowledge, the development of sinus bradycardia has not been described in patients with COVID-19 thus far.

Our case series included four patients; two males and two females. Two patients had pre-existing CV comorbidities including hypertension, coronary artery disease, and hyperlipidemia. All four patients developed sinus bradycardia during their ICU stay despite their CV state prior to admission. Their lowest pulse rates were 49, 46, 46, and 42 beats/min in patients 1-4 respectively. Bradycardia severity did not appear to be related to their pre-existing cardiac conditions. However, pre-existing CV disease and/or development of acute cardiac injury have been associated with significantly worse outcome in COVID patients [4, 7, 9-10].

The development of sinus bradycardia ranged from day four to day 15 of their hospitalization. This was transient with spontaneous resolution occurring within 24 hours to four days. Limited data are available on time course of development of cardiac manifestations in this infection. A study performed in China shows that increase in cardiac troponin I (cTnI) in fatal cases started around 16 days into their illness [11]. Reviewing our patients' cTnI values, all patients had cTnI <0.02 during bradycardia. Severe acute respiratory syndrome coronavirus (SARS-CoV) is another major viral respiratory tract infection which is of the same family of SARS-CoV-2, with a major outbreak in 2003. The pathological features in COVID-19 were found to resemble closely with those seen in disease seen with SARS-CoV [12]. Although not the most common manifestation, significant sinus bradycardia was seen in 14.9% of SARS-CoV patients, with a prevalence of 9.1%, 9.1%, and 4.4% in first, second, and third week of hospitalization, respectively. This was noted to be transient [7, 13], as seen in our patients. With onset of bradycardia ranging between four and 15 days of illness in these patients, time course for the development of bradycardia cannot be established at this point. More research requiring a larger sample size may help establish this.

Relative bradycardia is the term used to describe the mechanism where there is dissociation between pulse and temperature [14]. This has been reported in many infectious diseases including typhoid fever, Legionnaires’ disease, psittacosis, typhus, leptospirosis, malaria, babesiosis, and dengue fever [15-16]. We noted that during the bradycardia episodes our patients’ body temperatures had readings that went above 100 degree Fahrenheit. Although the term ‘relative bradycardia’ is used with body temperatures above 102 degree Fahrenheit [16], we believe this is still a significant finding due to the degree of bradycardia in our patients. This is an important finding to recognize as it provides insight to potential mechanisms of the disease process. The pathogenesis of relative bradycardia is poorly understood, but release of inflammatory cytokines, increased vagal tone, and direct pathogenic effect on the myocardium are few of the proposed mechanisms [17]. A group of researchers hypothesized that “relative bradycardia is the central mechanism reflecting and influenced potentially by the direct pathogenic effect on the sinoatrial node as well as cross-talk between the autonomic nervous system and immune system”. They further stated that cardiac pacemaker cells may be a target for inflammatory cytokines resulting in a change in heart rate dynamics or their responsiveness to neurotransmitters during systemic inflammation [14]. This is particularly interesting because recent studies show evidence of severe deterioration in some patients with COVID-19 being closely related to the cytokine storm [18]. Development of bradycardia may be a manifestation of this stage of the illness, implying the possible ‘calm before the storm’ in these patients. In a mouse model, it was noted that mice with bradyarrhythmia had increased levels of pro-inflammatory cytokines, including Interleukin (IL)-6, IL-10, IL-12, and tumor necrosis factor alpha (TNF-α) [19].

Potential mechanisms of bradycardia

The etiology of cardiac manifestations in COVID-19 patients seems to be multifactorial, which includes direct viral myocardial damage, hypoxia, hypotension, enhanced inflammatory status, angiotensin-converting enzyme 2 (ACE2)‐receptor downregulation, drug toxicity, and endogenous catecholamine adrenergic status [5, 7]. Direct myocardial injury from viral involvement of cardiomyocytes and the effect of systemic inflammation are thought to be the most common mechanisms responsible for cardiac injury [4, 9-10]. ECG changes in the severe stages of COVID-19 have been attributed to possible hypoxia and inflammatory damage incurred by the virus [3]. Our four patients had severe acute hypoxic respiratory failure, requiring intubation within 24 hours of hospital admission. Their inflammatory markers (ferritin, C-reactive protein, D-dimer, LDH, and fibrinogen) were elevated throughout bradycardic episodes which may imply a possible immunological damage leading to initial bradycardia. The inflammatory cytokines released during the stage of overwhelming immune response, acting on the cardiac pacemaker cells could possibly contribute to bradycardia. It may be that the high levels of pro-inflammatory cytokines, including IL-6 directly act on the sinoatrial (SA) node [14]. As all four patients developed bradycardia over six days into their illness, the time course falls within the timeline for onset of cytokine storm. This would be an interesting finding as it may be a sign of worsening inflammatory reactions or a prediction of cytokine storm. One may argue while all patients had elevated inflammatory markers during bradycardia, these did not appear to worsen on days following bradycardia episodes. In fact, in most patients, the inflammatory markers showed mild improvement. This may be due to early initiation of methyl-prednisone with or without tocilizumab in all patients, preventing severe inflammation.

As mentioned above, the pulse-temperature dissociation could imply a possible direct pathogenic effect on the SA node. A case report published in January 2011, reports sinus bradycardia on day seven of a patient with H1N1 infection. This was noted to be suggestive of “progressive involvement of conducting tissue and severity of disease, culminating into a fatal outcome” [20]. The group of researchers has also suggested that “in the terminal phase, SA node and AV node affection was preferentially more than the inter-nodal connecting pathways, bundle branch, or Purkinje fibers”.

Azithromycin and hydroxychloroquine are often used in the management protocol in COVID-19 patients who are hospitalized. These medications, especially when given together, are known to cause atrial and ventricular arrhythmias, and QTc prolongation [7, 9]. While patient 2 had initial prolongation of QTc prior to initiation of hydroxychloroquine, this improved while on the medication, and at the onset of bradycardia. All other patients developed bradycardia while on azithromycin and hydroxychloroquine combination, but had normal QTc intervals throughout bradycardia. We do not feel that the cause of bradycardia in our patients was due to medication-related QTc prolongation. Of note is that patients were on continuous infusion of propofol with or without dexmedetomidine during bradycardia episodes. While onset of bradycardia could have been related to initiation of propofol in patients 1 and 3, other patients were on propofol infusion three days prior to their episodes. Further, continuation, re-initiation, or rate increments of propofol or dexmedetomidine infusions after bradycardia resolution did not cause bradycardia. While there is always a chance of these medications inducing bradycardia, a clear correlation could not be found. There is also a potential for an exaggerated response of medication induced bradycardia in these patients, especially considering the severity of bradycardia.

## Conclusions

While cardiac manifestations have been reported and are now a recognized complication of COVID-19 pneumonia, transient sinus bradycardia has not been well described. SARS-CoV-2 viral infection appears to induce a transient sinus bradycardia, as noted in some patients with COVID-19. While etiology could be multifactorial, severe hypoxia, damage of cardiac pacemaker cells from inflammatory cytokines, and exaggerated response to medications are possible triggers. We believe it is important to know about the potential development of transient sinus bradycardia as a part of disease sequalae and for close CV surveillance of patients. A special consideration should be made in patients with inherited arrhythmia syndromes. It is particularly important to consider this to be a possible warning sign of a serious cytokine storm onset. As there are many medications being used empirically in patients, there is a need for increased awareness of possible drug interactions and close monitoring due to potential effects on AV conduction, QTc interval prolongation as well as worsening bradycardia. Bradycardia could be a possible predictor of worse outcome of COVID-19 as well. Further studies are needed to evaluate the prevalence of bradycardia occurring in COVID-19 patients, prognostic outcome in those who develop bradycardia, and long-term cardiac sequelae in survivors which is too early to assess at this point.
